# Social vulnerability and cardiovascular risk factors in adolescents

**DOI:** 10.1186/s12889-023-16959-z

**Published:** 2024-04-08

**Authors:** Viviane Freire de Farias, Larissa Almenara Soares, Luciana Nicolau Aranha, Ronir Raggio Luiz, Gláucia Maria Moraes de Oliveira, Glorimar Rosa

**Affiliations:** 1 Postgraduate Program in Medicine (Cardiology), Federal University of Rio de Janeiro, Rio de Janeiro, RJ Brazil; 2 Department of Nutrition and Dietetics, Josué de Castro Nutrition Institute, Federal University of Rio de Janeiro, Rio de Janeiro, RJ Brazil; 3 Institute for Studies in Collective Health, Federal University of Rio de Janeiro, Rio de Janeiro, RJ Brazil; 4 Universidade Federal do Rio de Janeiro, UFRJ, Cidade Universitária, R. Prof. Rodolpho P. Rocco, 255 – 8, Andar Sala 6, Rio de Janeiro, RJ Brazil

**Keywords:** Heart Disease risk factors, Social vulnerability, Food security, Social determinants of health

## Abstract

**Background:**

Social vulnerability can influence in the development of cardiovascular risk factors in adolescents (CRF). For this reason, the objective of our study was to evaluate the presence of CRF in adolescents, according to social vulnerability.

**Methods:**

This is a cross-sectional study with 517 adolescents of both sexes, from 10 to 19 years of age, classified into 2 groups by social vulnerability, according to socioeconomic characteristics collected by means of questionnaires, where adolescents who did not have access to drinking water, sewage network, and adequate per capita income were classified as vulnerable. Anthropometric, biochemical, and blood pressure data were evaluated. Level of physical activity was assessed by an adapted questionnaire, and food intake was assessed by a 3-day food record. Independent T, Mann-Whitney, and χ^2^ tests were used, according to the scale of measurement of the variables, on the statistical program SPSS, version 25, at a significance level of 5%.

**Results:**

Adolescents had median age of 14 (11 to 15) years; 58.4% were female; 32.4% were overweight, and 52.4% were physically inactive in leisure. Mean consumption of ultra-processed food was observed to account for 45.0% of calorie intake. Adolescents classified as vulnerable had lower weight, body mass index, waist circumference, hip circumference, and neck circumference when compared to non-vulnerable adolescents. Both groups had cholesterol concentrations above the normal level. Non-vulnerable adolescents had higher triglyceride concentrations, higher alcohol consumption, and lower fiber intake compared to vulnerable adolescents.

**Conclusions:**

Adolescents with social vulnerability are less likely to have cardiovascular risk factors.

**Supplementary Information:**

The online version contains supplementary material available at 10.1186/s12889-023-16959-z.

## Introduction

Premature exposure to cardiovascular risk factors (CRF) is associated with cardiovascular events in midlife. [1] Although most of the clinical manifestations of cardiovascular disease appear in adulthood, children and adolescents are affected by early exposure to these factors, showing an increasing trend toward dyslipidemia, obesity, and physical inactivity [1, 2]. Approximately 42 million children are overweight worldwide, at a ratio of 1 out of every 5. Obese children are 75% more likely to become obese adolescents, and 89% of obese adolescents may go on to become obese adults [3].

In Brazil, the Study of Cardiovascular Risks in Adolescents (ERICA, acronym in Portuguese) was conducted to evaluate the association of severity of obesity and cardiometabolic risk factors in a representative sample of Brazilian adolescents [4]. In a sub-sample of more than 37,000 adolescents, 17.2% were found to be overweight, 5.6% obese, and 1.3% severely obese. Furthermore, an increase was observed in the prevalence of high blood pressure, total cholesterol (TC) concentrations, low-density lipoprotein cholesterol (LDL-C), triglycerides (TG), glycated haemoglobin (HbA1c), blood glucose, insulin, metabolic syndrome, and low high – density lipoprotein cholesterol (HDL-C) concentrations, with the increase in the severity of obesity [4].

CRF, especially obesity, are strongly linked to positive energy imbalance related to inadequate nutrition and physical activity behavior, which are influenced by social vulnerability, such as low socioeconomic level, level of education, and housing conditions [5]. Studies have suggested that this is due to difficult access to economic resources and the living environment, reflecting that poorer neighborhoods have less opportunity for leisure and difficult access to a balanced diet [6].

Social vulnerability is a condition in which individuals who live in fragile situations or who lack access to rights such as income, adequate housing, water supply, basic sanitation, health services, schools, and quality public transport, among others, thus increasing susceptibility to damage that, eventually, results in social disconnection [7, 8]. Brazil is still one of the most unequal countries in the world, thus exposing adolescents to situations of high vulnerability [9].

Few studies have explored issues of socioeconomic conditions, social vulnerability, and CRF, especially in relation to adolescents. Understanding this relationship is important to understanding the way social vulnerability influences adolescents to develop CRF and then assist in public policies for this population. For this reason, the objective of our study was to evaluate the presence of CRF in adolescents, according to social vulnerability.

## Materials and methods

### Ethical aspects and study population

This is a cross-sectional study, which is part of research on the epidemiological profile of adolescents from public schools in the city of Arraial do Cabo, in the state of Rio de Janeiro, Brazil. Data collection was carried out in schools, with the support of a multidisciplinary team supervised by the study group. The sample size calculation was based on the G*Power3.1 program with effect size 0.19, β 0.80 and α 0.05, based on the article Van Hulst et al. 2015, with a total sample size of 220.

Adolescents, aged between 10 and 19 years old, of both sexes and any ethnicity, regularly enrolled in public schools in Arraial do Cabo in 2013 were included, and those who used any type of supplement, pregnant and lactating women, were excluded.

We obtained from this survey 517 eligible adolescents who filled in all the socioeconomic information and performed an anthropometric assessment so that they could answer the new question in this study. Of these, 260 underwent biochemical analysis, and 165 filled out the dietary data (Additional file 1).

This base study received approval in January 2013 from the Research Ethics Committee of the Clementino Fraga Filho University Hospital of the Federal University of Rio de Janeiro, under number 187.141, as well as from the Municipality of Arraial do Cabo. All parents or legal guardians of volunteers were duly informed about the procedures to which they would be submitted throughout the study, and they provided formalized consent by means of a free and informed consent form. All methods were performed in accordance with the relevant guidelines and regulations.

### Socioeconomic information and physical activity

An interview was conducted with parents and students to obtain socioeconomic, demographic, and health history information, by means of the application of a questionnaire. Smoking habits were categorized as non-smokers or smokers and former smokers were considered those who, after having been smokers, stopped smoking at least one month ago. To classify the results, the calculation of tobacco load was performed according to the number of cigarettes smoked per day divided by 20 (the number of cigarettes in a pack), and the result was multiplied by the number of years of tobacco use (years -pack) [10]. The consumption of alcoholic beverages was categorized as present or absent according to the Study of Cardiovascular Risks in Adolescents (ERICA) [11]. To assess physical activity, the study applied a questionnaire that had already been validated for the Brazilian population. This questionnaire allows the adolescent to inform the frequency (days) and time (hours and minutes) of the activities practiced. The product between time and frequency in each action was calculated to determine the level of physical activity, and the sum of the times obtained was calculated. Adolescents who did not accumulate at least 300 min per week of physical exercise were considered inactive in leisure [12].

### Social vulnerability

Social vulnerability was classified by means of data on the following 3 indicators: per capita income below 78.80 dollars, the cutoff point being 25% of Brazilian monthly minimum wage in the year 2013 (315.30 dollars); the presence of basic sanitation; and access to clean water. Adolescents who did not have any of these indicators were classified as socially vulnerable. There were thus two groups, one including socially vulnerable adolescents and one including non-vulnerable adolescents.

### Biochemical assessment

Biochemical analyses (glucose, total cholesterol, LDL-c, HDL-c, and triglycerides) were conducted by means of commercial kits (Wiener Lab. 2000, Rosario, Argentina), and reading was conducted using an A15 automatic analyzer (Wiener Lab. 2000, Rosario, Argentina). Fasting blood glucose was assessed using the glucose oxidase/peroxidase method. Values of 100 mg/dL or more were considered as high blood glucose. Triglycerides and total cholesterol were analyzed by the glycerol phosphate oxidase/peroxidase method; HDL-c was analyzed by the direct detergent method, and LDL-c was calculated following the Friedewald formula. The diagnosis of dyslipidemia was positive when there was an alteration in one or more serum lipid values by the Brazilian Society of Pediatrics (TC ≥ 170 mg / dL; LDL-c ≥ 110 mg / dL; HDL-c ≤ 45 mg / dL; TG ≥ 90 mg / dL) (Guide to dyslipidemia of the Brazilian Society of Pediatrics, 2020).

### Anthropometric assessment and blood pressure

Body mass and height were measured using a platform-type digital electronic scale with a stadiometer (Welmy®, São Paulo, Brazil), model W200A/1104, series number 775. Body Mass Index (BMI) / age Z-Score was calculated using Anthro Plus software, version 3.2.2 (World Health Organization, 2007) where adolescents with ≥ z-score + 1 and < z-score + 2 were classified as overweight and ≥ z-score + 2 as obese.

For circumference measurements, an inelastic measuring tape was used; the cutoff point for obesity according to waist circumference was the 90th percentile. Hip circunferente was measured, with the result expressed in centimeters. Neck circunferente ≥ 41 cm was considered high. The waist-to-hip and waist-to-height ratios were calculated by dividing waist/hip circumference and waist/height circumference, respectively. For the waist-to-height ratio, the cutoff point = 0.5 was used.

Blood pressure was measured using the oscillometric method with a cuff. Classification took age, sex, and height percentile into account. It was considered for ages 10 to 13 years, Normal blood pressure: percentile < 90, High blood pressure: percentile ≥ 90 to < 95; SAH stage 1: Percentile ≥ 95 to < 95 + 12mmHg and SAH stage 2: Percentile > p5 + 12mmHg. And for adolescents aged > 13 to 19 years, normal BP was considered: <120/<80mmHg; high BP: 120 to 129/<80mmHg; SAH stage 1: 130 to 139/80 to 89mmHg and SAH stage 2: ≥ 140/90mmHg, according to Guideline of the American Academic of Pediatrics, 2017.

### Dietary assessment

A 3-day food record was used to assess research participants’ dietary intake. With the help of the responsible person or a trained inspector, each volunteer filled in three records of alternate days, including a weekend or holiday, considering the average of the records. Meals consumed by students, offered by public schools (breakfast or snack and lunch), were considered. Data on energy, macronutrients (carbohydrates, proteins, and total lipids), fiber, and micronutrients were calculated using Food Processor software, version 7.2. The addition of 2 mL of soy oil to cooked or sautéed preparations and the addition of 10% sugar of the volume of liquids consumed were standardized, similar to the ERICA study [13]. The prevalence of micronutrient deficiencies was estimated by sex, age group, and proportion of adolescents with consumption below the estimated average requirement, as proposed by the Institute of Medicine of the United States in 2005 and validated in Brazil.

Classification of ultra-processed foods (UPF) was carried out in accordance with NOVA, based on the extent and purpose of applied food processing [14]. Group 4 of UPF was used in this study for detailed analysis of the amounts of energy, carbohydrates, proteins, total fats, saturated fats, dietary fiber, and sodium, which were calculated by Food Processor software, version 7.2. This analysis made it possible to verify the contribution of UPF to the total calorie value of the diet.

### Statistical analysis

Data are shown as absolute (n) and relative (%) frequency or median (25th to 75th percentile). The normality of the variables was investigated using the Kolmogorov-Smirnov test. The Independent T-test was used for variables with normal distribution; the Mann-Whitney test was used for variables with non-normal distribution, and, for categorical variables, a cross-tabulation table with χ^2^ test was used to compare the two groups. P values < 0.05 were considered statistically significant. Statistical analyses were performed using the statistical program SPSS, version 25.

## Results

Data were evaluated for 517 adolescents and 151 are in social vulnerability; 58.4% were female, and the median age was 14 years. More than half of the sample comprised adolescents who were inactive in leisure (52.4%) and non-White (54%), and median per capita income was 280.00 Brazilian reals. Social drinkers and smokers accounted for 12% and 0.8%, respectively.

The socioeconomic and demographic characteristics of the study groups are displayed in Table 1. The group of socially vulnerable adolescents showed significantly higher values for non-White individuals, lack of drinking water, and lack of sewage, as well as lower per capita income and lowest average age.

**Table 1:** Socioeconomic and demographic characteristics, by social vulnerability

Variables	Total (n = 517)	Non-vulnerable (n = 366)	Vulnerable (n = 151)	P value
**Age (years)**	14 (11–15)	14 (12–16)	12 (11–14)	**0.000**
**Boys % (n)**	41.6 (215)	43.7 (160)	36.4 (55)	0.126
**Girls % (n)**	58.4 (302)	56.3 (206)	63.6 (96)	
**Non-White % (n)**	54 (272)	51.1 (184)	61.1 (88)	**0.042**
**Per capita income ($)**	130 (79–210)	157 (116–232)	63 (46–81)	**0.000**
**Tobacco use % (n)**	0.8 (4)	0.8 (3)	0.7 (1)	0.856
**Alcohol consumption % (n)**	12 (60)	15.1 (53)	4.7 (7)	**0.001**
**Access to sewage % (n)**	13 (67)	0 (0)	44.4 (67)	**0.000**
**Access to running water % (n)**	5 (26)	0 (0)	17.2 (26)	**0.000**
**Inactive in leisure % (n)**	52.4 (271)	51.6 (189)	54.3 (82)	0.581
**Duration of physical exercise (minutes)**	260 (355–82.5)	280 (103.7–360)	200 (43.7–350)	0.223

Values expressed as median and interquartile range or frequency (n). T, Mann-Whitney, and chi-squared tests were applied, depending on the variable, to evaluate level of significance. Bold denotes values considered significant

Table 2 displays the anthropometric measurements and clinical data of the study groups; 32.4% of the adolescents had excess body mass. It was observed that the group of vulnerable adolescents had significantly lower values for body mass, height, BMI, waist circumference, hip circumference, and neck circumference, compared to the non-vulnerable group.

**Table 2:** Anthropometric and clinical data, by social vulnerability

Variables	Total (n = 517)	Non-vulnerable (n = 366)	Vulnerable (n = 151)	P value
**Weight (kg)**	52 (43–61.6)	53.8 (45.4–64.1)	46.8 (37–56.8)	**0.000**
**Height (cm)**	158 (150–165)	160 (152–167)	155 (145–161)	**0.000**
**BMI (Kg/m²)**	20.4 (18.1–23.8)	20.9 (18.6–24.2)	19.0 (16.9–21.7)	**0.000**
**Overweight% (n)**	32.4 (166)	34 (124)	28.6 (42)	0.238
**Waist circumference (cm)**	68 (63–76)	69 (63–78)	66 (60–71.7)	**0.000**
**Hip circumference (cm)**	88 (81–95)	89 (84–96)	85 (75–93)	**0.000**
**Waist-to-hip ratio**	0.78 (0.74–0.82)	0.78 (0.74–0.82)	0.79 (0.74–0.83)	0.432
**Waist-to-height ratio**	0.43 (0.40–0.48)	0.43 (0.40–0.48)	0.42 (0.39–0.46)	0.164
**Neck circumference (cm)**	31 (29–33)	31 (29–33.5)	30 (28–32)	**0.000**
**Normal BP % (n)** **High BP % (n)** **HAS Stage 1% (n)** **HAS Stage 2% (n)**	69.7 (350) 5.0 (25) 21.9 (110) 3.4 (17)	67.1 (237) 5.7 (20) 23.5 (83) 3.7 (13)	75.8 (113) 3.4 (5) 18.1 (27) 2.7 (4)	0,266

Values expressed as median and interquartile range or frequency (n). T, Mann-Whitney, and chi-squared tests were applied, depending on the variable, to evaluate level of significance. BMI: body mass index; BP: blood pressure. Bold denotes values considered significant

Table 3 displays borderline cholesterol values in both groups, presence of dyslipidemia in 178 adolescents (68.5%), and hyperglycemia in 21 adolescents (8.1%). The group of non-vulnerable adolescents had significantly higher triglyceride values, when compared to the non-vulnerable group.

**Table 3:** Biochemical data, by social vulnerability

Variables	Total (n = 260)	Non-vulnerable (n = 168)	Vulnerable (n = 92)	P value
**Fasting blood glucose (mg/dL)**	84 (77–89)	84 (79–90)	80 (76–87.7)	0.128
**Cholesterol (mg/dL)**	157 (140–174)	157.5 (142–173)	154.5 (138–175)	0.102
**Triglycerides (mg/dL)**	78 (62–95)	80.5 (64.2–96)	73.5 (56.5–89.7)	**0.027**
**HDL-c (mg/dL)**	47 (42–50)	47 (42–50)	48 (43–50)	0.569
**LDL-c (mg/dL)**	93 (78.2–110)	93 (78.2–111.7)	92.5 (77.1–109)	0.229
**Hyperglycemia % (n)**	8.1 (21)	8.3 (14)	7.6 (7)	0.383
**Dyslipidemia % (n)**	68.5 (178)	69.6 (117)	66.3 (61)	0.580

Values expressed as median and interquartile range or frequency (n). T, Mann-Whitney, and chi-squared tests were applied, depending on the variable, to evaluate level of significance. HDL-c: high-density lipoprotein cholesterol; LDL-c: low-density lipoprotein cholesterol. Bold denotes values considered significant

According to dietary analysis, as displayed in Table 4, we observed a high consumption of UPF in all the adolescents studied (45.0%). There was low consumption of fiber, calcium, vitamin D, and omega 3 on the part of the entire study population. According to the classification of vulnerability, there is a statistically significant difference between groups by food intake; the group of vulnerable adolescents showed lower calorie and vitamin D intake and higher sodium and calcium intake. The group of non-vulnerable adolescents had higher total energy value, higher caloric intake, and lower omega 3 and fiber intake.

**Table 4:** Dietary data, by social vulnerability

Variables	Total (n = 165)	Non-vulnerable (n = 108)	Vulnerable (n = 57)	P value
**TEI (Kcal)**	1991 (1889.7–2298.5)	1991 (1966.9–2396.3)	1889.7 (1761.7–1978.9)	**0.000**
**ETEV (Kcal)**	1837.7 (1662.4–2093.8)	1853.4 (1681.6–2109.7)	1767.3 (1628.9–2009.4)	**0.023**
**UPF (% of TEI)**	45.0 (25.4–59.1)	45.3 (25.6–58.7)	43.9 (21–62.2)	0.614
**Proteins (g)**	84.9 (65.7–104.9)	84.3 (63.8–104.7)	87.2 (72.4–105.4)	0.729
**Carbohydrates (g)**	258.7 (203.3–309.8)	245.7 (194.9–302.9)	272.2 (233.6–327.2)	0.057
**Fiber (g)**	23.4 (16.2–31.0)	21.3 (14.4–29.0)	27 (22.4–32.7)	**0.004**
**Sugar**	91.5 (54.6–117.3)	85.3 (49.0–110.6)	95.8 (62.7–130.6)	0.119
**Sodium (mg)**	1548.4 (784.5–2278.5)	1284.7 (110.6–2147.3)	1820.9 (1336.6–2322.4)	**0.005**
**Total lipids**	52.5 (41.7–70.6)	53 (42.5–70.4)	50.9 (41.3–71.1)	0.970
**Omega 3 FA (g)**	0.5 (0.1–0.7)	0.46 (0.02–0.69)	0.59 (0.46–0.76)	**0.000**
**Calcium (mg)**	17.9 (7.8–73.6)	351.7 (4.6–556.1)	478.2 (377.9–649.0)	**0.002**
**Vitamin D (mcg)**	3.0 (1.8–6.4)	3.6 (2.0–28.9)	2.6 (1.4–3.6)	**0.000**

Values expressed as median and interquartile range or frequency (n). T, Mann-Whitney, and chi-squared tests were applied, depending on the variable, to evaluate level of significance. ETEV: estimated total energy value; FA: fatty acids; Kcal: kilocalories; TEI: total energy intake; UPF: ultra-processed foods. Bold denotes values considered significant

## Discussion

This study has examined how socioeconomic adversity can affect adolescents. The results have provided evidence that there is a difference between adolescents with social vulnerability; they were shown to be less prone to CRF, differing from the evidence that most studies have provided [15, 16]. This difference may be attributed to low per capita income, leading to scarce access to food, with the main food source being school meals, which have healthier and more balanced options, when compared to the type of food consumed by vulnerable adolescents commonly published in literature from outside Brazil [7, 17]. In general, the majority of vulnerable adolescents in this study were non-White, and they showed lower anthropometric measurements compared to non-vulnerable adolescents. The reality of Brazil’s economic and social structure constitutes one of the highest levels of income inequality worldwide [9]. This status of vulnerability hinders adequate access to safe and nutritious foods, which may increase the prevalence of food and nutritional insecurity. [7].

A prospective study by IGUACEL et al., in 2018, [8] evaluated more than 8000 children between 2 and 9 years of age, from 8 European countries and re-evaluated them 2 years later, classifying 4 groups of social vulnerability. In the group whose parents did not have a social support network, it was observed that children were more likely to be thinner than non-vulnerable children; however, the other vulnerable groups showed associations with overweight and obesity. In our study, vulnerable adolescents had lower weight, BMI, waist circumference, hip circumference, and neck circumference, when compared to non-vulnerable adolescents, which may be explained by their age and low-calorie intake. These results were different from those observed by other researchers who provided evidence that socially vulnerable adolescents from Liverpool and Chile showed higher rates of overweight and obesity. [6, 18] The Brazilian population of adolescents has different characteristics than adolescents from high-income countries, where most studies have been conducted. [17].

In their 2020 study, IGUACEL et al. [19] observed that European adolescents whose mothers had low levels of education or who had more than three socioeconomic disadvantages were at a higher risk of metabolic syndrome. A cohort study conducted in Finland observed that children and adolescents living in socioeconomically disadvantaged areas had greater health risks, evolving into adulthood with risk of altered glucose metabolism; by middle age, cumulative socioeconomic disadvantage was associated with increased CRF and diabetes incidence [20].

In the total sample, 32.4% were overweight, with no significant difference between groups; however, the group of non-vulnerable adolescents had a higher percentage of overweight, and this was greater than the result found in the ERICA study, which provided evidence that 25.5% of Brazilian adolescents were overweight. [21] According to CARDEAL et al. (2020), [5] obesity is a public health problem, especially in high-income countries, where more than 20% of children are obese; obesity is strongly related to low socioeconomic status, and it differs by race and ethnicity.

Regarding physical activity, we did not observe any difference between vulnerable and non-vulnerable adolescents, showing that physical inactivity and sedentary behavior are risk factors for adolescents of all socioeconomic levels. Various studies have demonstrated, especially in the early stages of life, the importance of physical exercise, given that it can promote better school performance and maintenance of blood glucose concentrations, lipid profile, and blood pressure, with consequent reductions in diabetes, obesity, and hypertension, respectively [22].

Another important finding was alcohol consumption in the total sample was 12%, which is lower than that of 24.2% found in the ERICA study [23]. The percentage of participants who used alcohol was significantly in the group of non-vulnerable adolescents (15.1%), when compared to the vulnerable group (4.6%).

The National School Health Survey (PeNSE, acronym in Portuguese) conducted in 2015 demonstrated that 54.3% of adolescents in the age range between 13 and 15 years had already tried a dose of an alcoholic beverage, while 73% of students 16 and 17 years old had also already consumed alcoholic beverages. [24] Consumption of large quantities of alcohol has been indicated as a risk factor for high blood pressure, obesity, and stroke, among other cardiovascular diseases. [23].

Both our study groups had total cholesterol concentrations above normal (157 mg/dL), which may be explained by the high consumption of UPF [25]. We observed more than half of the studied population had dyslipidemia and significant differences between groups for triglyceride concentrations, where non-vulnerable adolescents had higher triglyceride concentrations when compared to the group of vulnerable adolescents. This may be related to higher alcohol intake, higher UPF consumption, and lower levels of physical exercise [26].

We observed that the adolescents studied had a high consumption of UPF, accounting for 45% of the calories ingested, differing from the result of the POF 2017–2018 survey, which showed that UPF provided about 27% of the total daily energy of Brazilian adolescents. [27] Exposure to this type of food includes high amounts of sodium, saturated fat, trans fat, and sugar, as well as low intake of fiber, minerals, and vitamins that are present in whole grains, fruits, vegetables, nuts, seeds, and fish. [28].

Studies have provided evidence that UPF consumption is directly associated with the risk of non-communicable chronic diseases, such as obesity, diabetes, hypertension, dyslipidemia, cardiovascular and cerebrovascular diseases, cancer in general and depression, in addition to early mortality due to any cause. [29].

A study based on the ERICA database, with more than 71,000 Brazilian adolescents, aimed to observe their dietary patterns by national region, and they observed that, in all regions, male adolescents reported greater adherence to the traditional pattern, with a healthier diet, while adolescent students from private schools and those with higher socioeconomic level were associated with consumption of unhealthy foods such as sugary drinks and snacks [30]. A study by AZEMATI et al. (2018) [31] provided evidence that Iranian children and adolescents who consumed junk food showed higher likelihood of being overweight and having high blood pressure.

The results of this study indicate that there are differences in the dietary patterns of the adolescents studied when compared to the recommendations and to vulnerable adolescents. This finding can be explained by the presence of the National School Feeding Program (PNAE, acronym in Portuguese) in public schools, which aims to ensure healthy and adequate food [30]. Vulnerable adolescents eat their main meals at school, while non-vulnerable ones have higher access to an unhealthy, industrialized diet outside the school environment, due to greater income availability [17].

The main limitations to this study are that it is based on self-reported data and logistical difficulties in carrying out the food record and blood collection, due to refusal, similar to what has been observed in studies conducted in school environments [23]. In spite of these limitations, it is important to underscore that this is the first study to assess CRF, by means of biochemical markers and anthropometric assessment, in adolescents and UPF consumption according to social vulnerability in Brazil. With that in mind, social vulnerability and UPF consumption is currently a highly debated topic, and studies on these topics are scarce in adolescents.

There were different definitions for the concept of social vulnerability. Several studies described vulnerability, focusing on the subject’s social vulnerability. In other studies, vulnerability was linked to demographic and socioeconomic characteristics of the population, health conditions, access to essential services, deprivation of rights, and shortage of material resources. Also, some authors consider a multiplicity of overlapping factors in the perspective of social vulnerability: socioeconomic conditions, access to services, culture prevalent, social relations, and subjectivity [32]. We used the three indicators: per capita income below 78.80 dollars, the cutoff point being 25% of Brazilian monthly minimum wage in the year 2013 (315.30 dollars); the presence of basic sanitation; and access to clean water that was available in our database trying the concept to social vulnerability as an imbalance between material and symbolic resources available to the subject and your needs. It may be a limitation of our study, but the concept of social vulnerability isn’t well defined and varied in various studies, as discussed above.

Accordingly, it is important to emphasize that the data from this study can be applied to the school environment in planning strategies aimed at promoting and raising awareness regarding food access among students. Due to the social vulnerability of children and adolescents who study in public schools in Brazil and who have school lunches as their main meal, it is essential to promote strategies to improve access to healthy foods in the school environment. New studies need to be conducted to investigate the impact of school lunches on reducing dietary risks in Brazilian children and adolescents.

## Conclusion

In conclusion, this study has provided evidence that vulnerable adolescents have lower energy consumption, body mass, and anthropometric measurements, when compared to non-vulnerable adolescents, who may in turn be more prone to CRF.

### Electronic supplementary material

Below is the link to the electronic supplementary material.

Additional file 1: Flowchart of the adolescent’s participation

## Data Availability

All data generated or analysed during this study are included in this published article ans its supplementary files.

## Abbreviations

**BMI**: body mass index
**BP**: blood pressure
**CRF**: cardiovascular risk factors
**ERICA (acronym in Portuguese)**: Study of Cardiovascular Risk in Adolescents
**ETEV**: estimated total energy value
**FA**: fatty acids
**HBA1c**: glycated haemoglobin
**HDL-c**: high-density lipoprotein cholesterol
**Kcal**: kilocalories
**LDL-c**: low-density lipoprotein cholesterol
**PeNSE (acronym in Portuguese)**: National School Health Survey
**PNAE (acronym in Portuguese)**: National School Feeding Program
**TC**: total cholesterol
**TEI**: total energy intake
**TG**: triglycerides
**UPF**: ultra-processed foods
