# Supplementary material for: Social vulnerability and cardiovascular risk factors in adolescents
Source: BMC Public Health. 2024 Apr 8;24:982. doi: 10.1186/s12889-023-16959-z (PMC11000392; doi:10.1186/s12889-023-16959-z)
Supplement: Supplementary file 1 — Additional file 1: Flowchart of the adolescent’s participation [file 12889_2023_16959_MOESM1_ESM.docx]

**Additional file 1.** Flowchart of the adolescent’s participation

**Vulnerable**

**(n= 92)**

**Non-vulnerable**

**(n= 168)**

Underwent biochemical analysis

(n=260)

Completely filled out the dietary data

(n=165)

**Vulnerable**

**(n= 57)**

**Non-vulnerable**

**(n= 108)**

Did not complete food records

(n=352)

Refused blood collection

(n=257)

Eligible adolescents

(n=517)

Did not fill in all socioeconomic data

(n=1429)

Did not accept to participate in the study

(n=54)

Recruited adolescents

(n=2000)
